# Contrast-enhanced and microvascular ultrasound imaging features of testicular lymphoma: report of five cases and review literature

**DOI:** 10.1186/s12894-022-00957-1

**Published:** 2022-01-24

**Authors:** Li Yang, Yuan Tao, Zhang Weixin, Bao Meiling, Hang Jing

**Affiliations:** 1 Department of Urology, The First Affiliated Hospital of Nanjing Medical University, Nanjing, 210029 China; 2 Department of Ultrasound, The First Affiliated Hospital of Nanjing Medical University, Nanjing, 210029 China; 3 Department of Pathology, The First Affiliated Hospital of Nanjing Medical University, Nanjing, 210029 China

**Keywords:** Lymphoma, Contrast-enhanced ultrasound, Microvascular ultrasound, Testicle

## Abstract

**Background:**

To retrospectively investigate the grey-scale, Doppler, contrast-enhanced and microvascular ultrasound of five patients with primary testicular lymphoma of our institute through review literature analysis.

**Methods:**

From January to November 2020, five patients with primary testicular lymphoma confirmed by histology were preoperatively investigated with a standardized sonographic protocol including contrast-enhanced and microvascular ultrasound.

**Results:**

Conventional ultrasound showed localized hypoechogenicity represented with solitary (2 of 5), multiple lesions (2 of 5), or entire testicular involvement (1 of 5). Increased blood flow appeared in color Doppler ultrasound with straight vascular sign (4 of 5). In contrast-enhanced ultrasound images confirmed this pattern (4 of 5) and presented hyper enhancement with enlarged range. On microvascular ultrasound imagings, all lesions were presented with straight and parallel course of intralesional vessels (5 of 5).

**Conclusions:**

Here, we identified an increased vascularity with enlarged range on contrast-enhanced ultrasound along with a linear nonbranching pattern by vascular sign on microvascular ultrasonographic of testicular lymphoma.

## Introduction

Primary testicular lymphoma (PTL) is a malignant tumor occurs in an immune-privileged site [1], which is a rare extra-nodal non-Hodgkin's lymphoma (NHL), accounts for approximately 9% of testicular tumors [2, 3]. PTL is the most common bilateral testicular tumor and 6–15% of them have simultaneous bilateral involvement [4]. The majority sub type of PTL is diffuse large B-cell lymphoma (DLBCL), rare sub types are mantle-cell, NK/T-cell and other T-cell lymphomas [2, 5, 6]. As an invasive malignant tumor with progression-free and low survival rate [2], PTL often occurs in the elderly and the median diagnosis age is 67 [7]. Positron emission tomography-computed tomography (PET-CT) is considered to be the standard practice for both staging and response evaluation [5]. The treatment of PTL consists of orchiectomy followed by immunochemotherapy, irradiation or excision of the contralateral testis due to the central nervous system prophylaxis [8]. Orchidectomy provides a therapeutic advantage, as it may confer a therapeutic advantage by gaining better local control and removing a possible sanctuary site for relapse [9].

If there was a history of testicular swelling or clinical evidence of testicular mass or enlargement, ultrasonography (US) was recommended. For PTL lesions, conventional US presented a specific appearance with focal and diffuse hypoechoic like a ‘moon on the water’ [10]. However, images were difficult to distinguish it from other types of testicular neoplasms [11]. Grayscale and color Doppler flow image (CDFI) reported the appearance of well-defined homogeneous hypoechoic lesions with marked hypervascularization [12, 13]. Contrast-enhanced ultrasound (CEUS) improved the sensitivity of intensifying the vasculature visibility and had recently expanded to evaluate testicular lesions [14]. Furthermore, CEUS was also introduced into a routine preoperative workup of testicular surgery patients [15, 16]. And a rapid filling time (< 7 s) and straight vessel pattern were reported as the sonographic hallmarks in CEUS in PTL lesions [17]. A recent study based on multiparametric sonography found that PTL was characterized by increased blood vessels on CDFI and CEUS along with the increased stiffness on strain elastography [18].

Microvascular ultrasound, a latest development of color Doppler technology, is a non-invasive microvascular imaging technique using low flow Doppler signal processing [19]. Slow and fine vascular flows had possibly precise detected using microvascular US [20, 21]. So far, no paper was published on microvascular US image in PTL. Therefore, we reported the CEUS and microvascular US features of five cases of PTL, along with summarizing the US, CEUS and microvascular features of the tumor.

## Materials and methods

### Patient selection

The researchers retrospectively analyzed images of patients with testicular tumors who underwent conventional US, CDFI, CEUS and microvascular ultrasound in the department of ultrasound and urology of the First Affiliated Hospital of Nanjing Medical University between January to November 2020. The study was approved by the Ethics Committee of the First Affiliated Hospital of Nanjing Medical University (Nanjing, China) and performed according to the Helsinki Declaration. Informed consent was waived. All the participants gave their informed consent for the publication of their images in an online open-access publication. The enrolled clinical information and laboratory data such as clinical history, tumor side, swelling or pain were also recorded in detail. According to the immunohistochemical results, confirmed diagnosis of PTL cases and the corresponding images were enrolled.

### US, CEUS and microvascular US examination of testis

Two doctors with more than 10 years' experience (L.Y. and Y.T.) in testicular US performed the examinations included in this study. Gray-scale US, CDFI and CEUS were performed using a real-time US device (Philips Healthcare, Eindhoven, the Netherlands) equipped with a 6–15 MHz linear array transducer. Microvascular US image was collected using a real-time US device (Aixplorer, SuperSonic Imagine, Aix-en-Provence, France) equipped with a 4–15 MHz linear array transducer. CEUS examinations were also performed by the same transducer at a low mechanical index (0.08), focus positioned behind the region of interest after the injection of 2.4 mL of the sulfur hexafluoride contrast agent SonoVue (Bracco SpA, Milan, Italy), followed by 10 mL of 0.9% saline. When the microvascular US was activated, the transducer was placed on the surface of scrotum without compression or movement.

### Sonographic interpretation

The doctors examined the conventional US images first and focused on homogeneity, size, echogenicity (compared with surrounding normal testicular tissue), boundary, margin, extent of infiltration, calcification and presence of the formerly described “straight vessel [12]” or “linear echogenic strands [22]” pattern of testicular mass. The tumor’s flow grade [23], velocity, resistance index (RI) and exist of “straight vessel pattern” were also recorded during the CDFI evaluation.

CEUS was performed on the lesions including normal testicular tissue and at least more than 50% of the tumor area. During the CEUS examination, probe was placed on the skin surface of scrotum without movement, and the dynamic images were observed more than 2 min after SonoVue injection. The CEUS evaluation was analyzed by two doctors with more than 10 years working experience in CEUS (L.Y. and H.J.). Size change was compared to the same section in grey-scale US at peak time in CEUS. Time-intensity curve (TIC) analysis was performed for evidence of the degree of overall enhancement (microperfusion) within the target lesions. And peak intensity (PI), time to peak (TTP), and rise time of tumor and surrounding normal testicular tissue were recorded. According to TIC results, we classified increased, decreased, or similar enhancement patterns of the mass (compared with the intensity of surrounding testicular tissue or contralateral testis). Boundary (clear or unclear), size change and homogeneity were also assessed. Furthermore, we also focused on the presence of formerly described “linear nonbranching [18]” pattern in CEUS images.

The microvascular US was assessed for evidence of the degree of overall enhancement (microperfusion) within the target lesions, which was characterized as increased, decreased, or similar to normal parenchyma, and the macrovascular pattern was described as linear nonbranching or random similar to CEUS.

### Histopathology and immunophenotype

The enrolled five cases were confirmed by immunohistochemical pathological results. All specimens were fixed with 3.7% neutral formaldehyde solution, routinely dehydrated, embedded in paraffin, 3um thick sections, HE and immunohistochemical staining. Immunohistochemical staining: En Vision two-step method was used, and negative and positive controls were also set up. All primary antibodies (EBER, CD20, PAX5) were purchased from Fuzhou Maixin Biotechnology Co., Ltd., and TBS was used as negative control instead of primary antibody.

## Results

### Clinical features and histological findings

We reviewed and analyzed the clinical and US data of 18 patients with testicular mass in this study. According to pathological immunohistochemistry results, images of 6 patients with PTL were selected. One case was excluded because of incomplete imaging data. Finally, US and CEUS images of 5 patients were included. The baseline clinical characteristics of the enrolled cases were shown in Table 1. The median age of the selected patients was 64 (range 31–91). All the enrolled patients had no cryptorchidism, hypospadias, inguinal lymph node enlargement and lumbago. Three cases were painless and two cases were complicated with testicular swelling and pain. The average clinical history was 1.60 ± 0.89 months, and average diameter was 46.3 ± 10.9 mm. Four patients were DLBCL and one patient was extra-nodal NK/T cell lymphoma.

**Table 1:** Clinical and ultrasound features of testicular cases in this series

	Patient	1	2	3	4	5
Clinical features	Age	64	91	46	31	65
	Tumor side	L	R	R	R	R
	Pathological result	DLBCL	DLBCL	DLBCL	NK/T cell lymphoma	DLBCL
Grey-scale US	Size(mm)	35	37	48.5	62	49
	Extra-testicular findings	No	Yes	No	Yes	Yes
	Straight vessel	Yes	Yes	No	Yes	Yes
CDFI	Straight vessel	Yes	Yes	No	Yes	Yes
	Flow grade	III	III	III	III	III
CEUS	Size change	enlarged	enlarged	enlarged	enlarged	enlarged
	Boundary	unclear	unclear	unclear	unclear	unclear
	PI(dB)	4.36	9.68	3.32	10.43	17.53
	TTP(s)	27.19	44.48	35.13	22.18	30.07
	Rise time(s)	5.34	8.70	9.13	4.35	0.51
	Linear nonbranching	Yes	Yes	No	Yes	Yes
Microvascular US	Flow degree	Increased	Increased	Increased	Increased	Increased
	Pattern	Linear	Linear	Linear	Linear	Linear

*L* left, *R* right, *NK/T* cell lymphoma, extra-nodal natural killer/T-cell lymphoma

### US, CEUS and microvascular US features

**Fig. 1:**
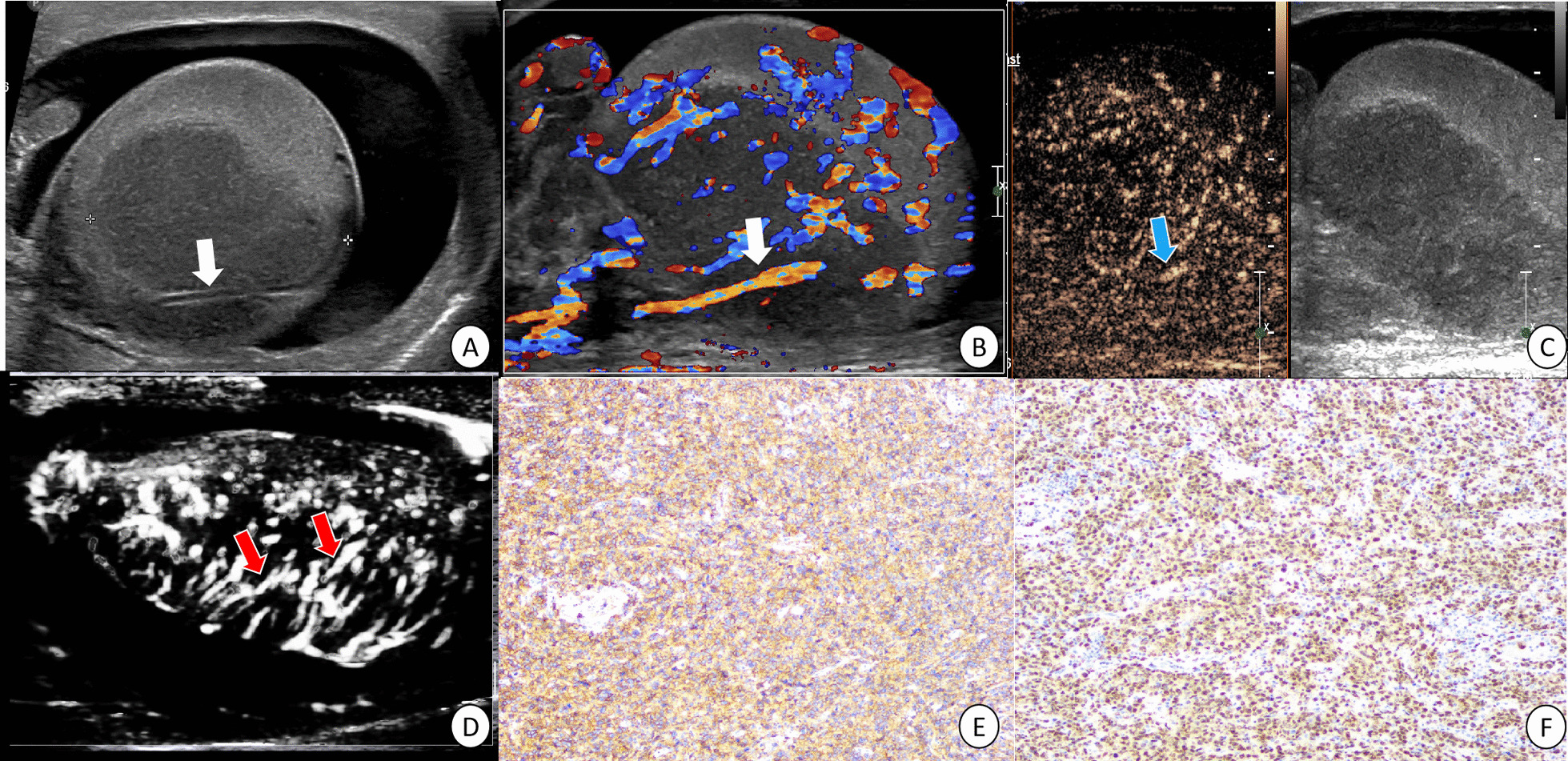
A diffuse large B-cell lymphoma of right testis in a 91-year-old patient proven after orchiectomy. White arrows showed the straight vessel pattern in grey-scale US (**A**) and CDFI (**B**) image. The lesion showed increased enhancement with enlarged range on contrast-enhanced ultrasound (**C**). Blue arrow presented the linear nonbranching in CEUS image (**C**). Red arrows showed linear nonbranching pattern in microvascular US image (**D**). Tumor cells were positive for CD20(+) (**E**) and PAX5(+) (**G**) by immunohistochemistry

**Fig. 2:**
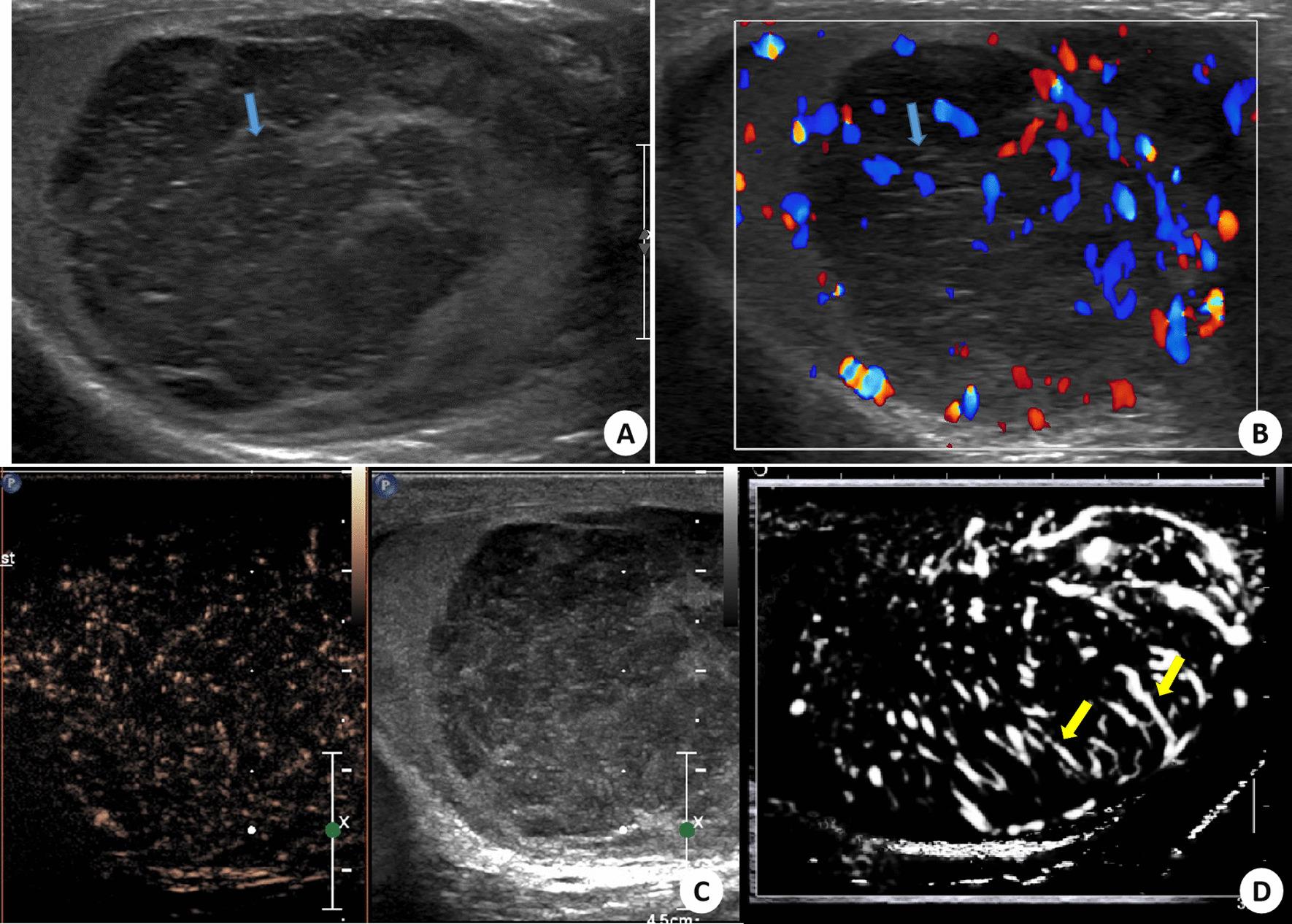
A diffuse large B-cell lymphoma of right testis in a 46-year-old patient diagnosed after orchiectomy. Blue arrow indicated the linear echogenic strands in grey-scale US (**A**) and CDFI (**B**) images. The increased enhancement with enlarged range was also seen in CEUS image (**C**). Yellow arrows (**D**) indicated the straight and parallel vascular sign on microvascular ultrasonographic image

**Fig. 3:**
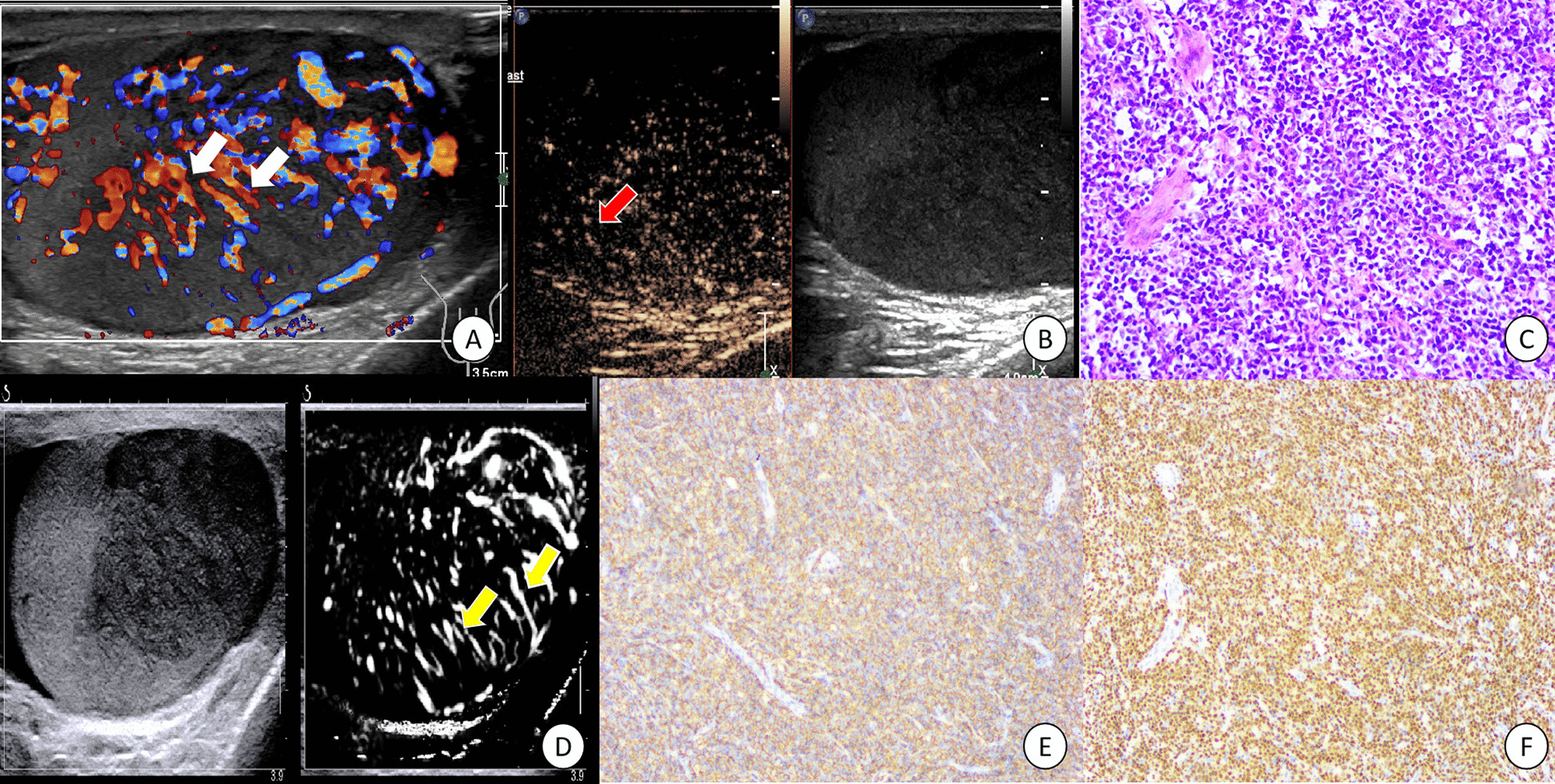
A diffuse large B-cell lymphoma of left testis in a 64-year-old patient. White arrows indicated the straight vessel pattern in CDFI image (**A**). Red arrow showed the linear nonbranching pattern in CEUS image (**B**). In HE*200 (**C**), medium and large lymphocytes were diffusely distributed. Yellow arrows (**D**) indicated the straight and parallel vascular sign on microvascular US image. Tumor cells were positive for CD20(+) (**E**) and PAX5(+) (**F**) by immunohistochemistry

**Fig. 4:**
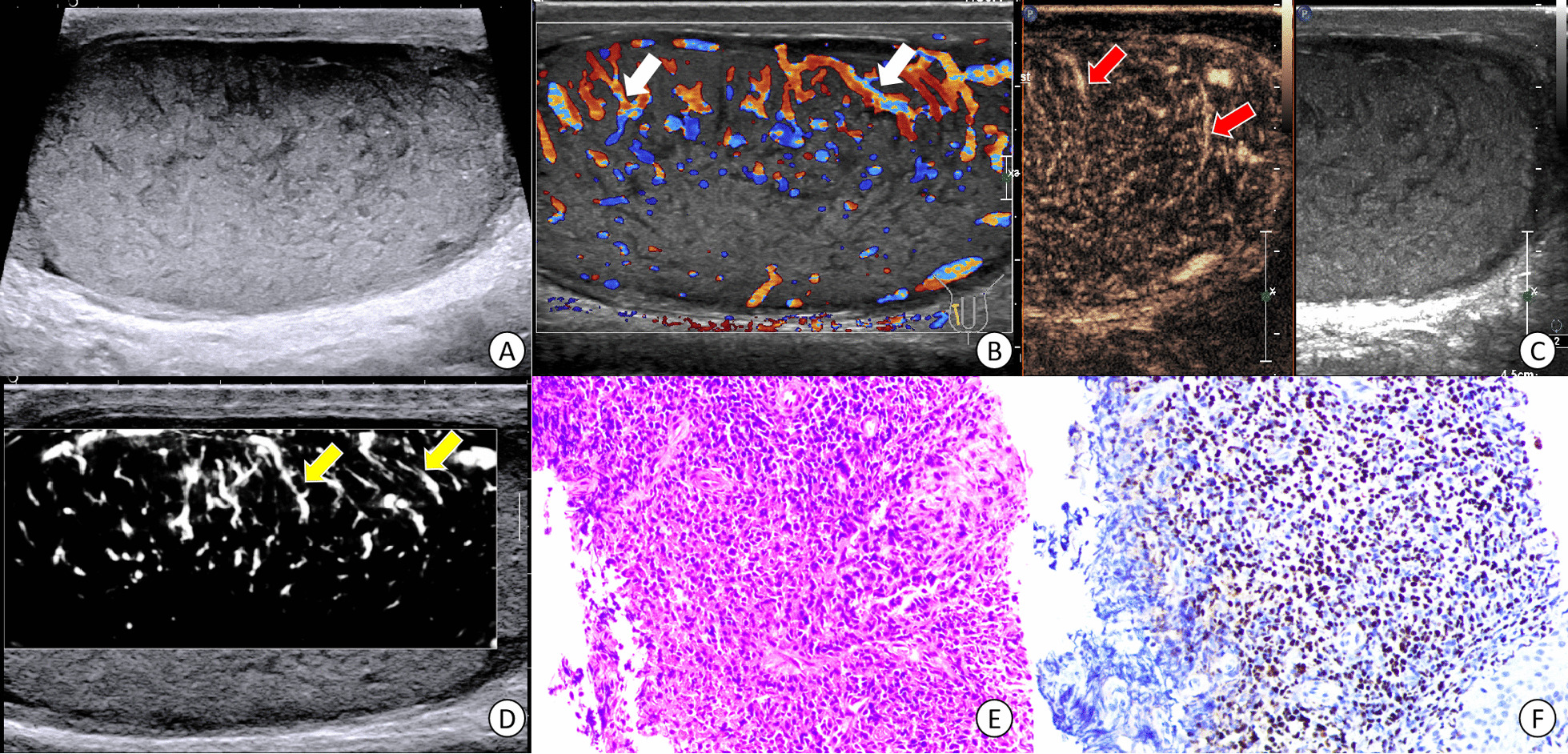
Images from a 31-year-old patient with histological proven extra-nodal NK/T cell lymphoma. Because of the poor cardiopulmonary function, he received a core needle biopsy of right testis due to recurrence. Multiple diffuse hypoechoic lesions were detected and the straight vessel pattern was seen in grey-scale US (**A**). White arrows indicated the straight vessel pattern in CDFI image (**B**). Red arrows showed the linear nonbranching pattern in CEUS image (**C**). Yellow arrows (**D**) indicated the straight and parallel vascular sign on microvascular US image. Diffuse distributed lymphocytes were seen in HE*200 (**E**). Tumor cells were positive for EBER(+) (**F**) by immunohistochemistry

In gray-scale US images, all the enrolled patients showed irregular solid hypoechoic testicular lesions without calcification or cystic degeneration. Four lesions were located in testis and one lesion involved extra-testicular, including ipsilateral epididymal head and spermatic cord. Straight vessel pattern were showed in 4 of 5 (Figs. 1, 3 and 4) and linear echogenic strands appeared in 1 of 5 (Fig. 2). Diffuse infiltration of the entire testis in 2 of 5 and homogeneous hypoechoic in 4 of 5. In CDFI images, five lesions presented marked hypervascularization with flow grade III. Straight blood vessel sign in 4 of 5 (Figs. 1, 3 and 4).

According to TIC analysis, five PTL lesions showed hyper enhancement pattern in CEUS (Figs. 1, 2, 3 and 4). Among the quantitative parameters, PI (9.06 ± 5.68 dB) of PTL was higher than normal testes (3.43 ± 1.95 dB), but TTP (31.81 ± 8.50 s) was shorter than normal testes (38.01 ± 11.25 s). All the enrolled lesions showed a rapid hyper enhancement with enlarged range and unclear boundary in CEUS. Of most cases (4 of 5), a linear nonbranching pattern was seen (Figs. 1, 3 and 4). On microvascular US images, all the lesions presented an increased flow with linear nonbranching pattern (Figs. 1, 2, 3 and 4). The detailed US data of the 5 PTL lesions were shown in Table1.

### Pathological and histological results

Grossly, the resected testicular lesions showed white or brown solid masses on the section, with size ranging from 3 to 6 cm. Histopathologically, the tumor had a diffuse growth pattern, mainly involving interstitial tissue, surrounded by atrophic seminiferous tubules. The tumor cells of PTL showed an obvious vascular centrality and vascular invasive growth pattern. In one case, invasion of epididymis and spermatic cord was found. All the five cases were diagnosed by immunohistochemical examination. One case was extra-nodal NK/T cell lymphoma (EBER+), and the other four cases were DLBCL (CD20+, PAX5+).

## Discussion

PTL is a rare malignant hematological tumor, and its treatment is different from other testicular tumors [24]. In this report, we retrospectively analyzed the CEUS and microvascular US features of five cases of PTL. We revealed that the PTL lesions presented by a hyper enhancement by enlarged range in CEUS along with a nonbranching linear vascular pattern on microvascular US. Compared to normal testicular tissue, PTL showed a rapid hyper enhancement mode. After combination of the focal hypoechoic, hypervascularization with straight vessel pattern in US, PTL could be indicated.

So far, there had seven reports about CEUS features of PTL [16–18, 25–27], along with our report. In these papers, a image characteristic of hyper enhancement along with linear nonbranching sign was proposed. Table 2 summarized the detailed clinical and ultrasonic features of these reports.

**Table 2:** Summary of US and CEUS features of PTL cases

References	Case number	Tumor side	CDFI	CEUS	Microvascular US
Shu et al. [25]	1	L	–	Hyper enhancement	–
Guntram et al. [16]	1	–	–	Hyper enhancement	–
Guntram et al. [17]	6	–	Marked hypervascularization	Straight vessel patter, rapid filling time	–
Kachramanoglou et al. [18]	8	1B/4R/3L	Straight vessel pattern	Linear nonbranching	–
Schwarze et al. [26]	3	1B/2L	–	Hyper enhancement	–
Jie et al. [27]	8	–	Hypervascularization	Hyper enhancement, linear nonbranching	–
This report	5	4R/1L	Straight vessel pattern	Hyper enhancement with enlarged range, linear nonbranching	Linear nonbranching

*L* left, *R* right, *B* bilateral

The pathological changes of PTL were different from other testicular tumors. It was characterized by tumor cells surrounding and compressing seminiferous tubules and normal testicular vessels, involving interstitial tissue [2]. This pathological characteristic might be the reason for the appearance of straight vessel sign in US or CDFI images. However, this feature was not unique to PTL. In other invasive tumors (such as plasmacytoma or leukemic infiltration) and non-neoplastic diseases (such as chronic inflammatory diseases), the similar sonographic findings might also be appeared. In this report, the straight vessel pattern was also seen on grey-scale and CDFI images. This result was consistent with those of previous reports and demonstrated that US had high repeatability and reliability in the evaluation of testicular lymphoma. In our report, a linear echogenic strands pattern, as reported in the previous research, was presented in one case. This sign was similar to the previously reported thyroid DLBCL images [22]. These fibrous bands separated the tumor body into a map shape, which indirectly reflected the pathological process of tumor tissue infiltrating growth and fibrous connective tissue proliferation encircling repeated antagonism.

CEUS improved the characteristics of testicular lesions and could evaluate the microvascular information in detail [16]. Tenuta et al. reported that compared with other testicular malignancies, PTL had a higher degree of enhancement [28]. As we know, quantitative analysis can provide more objective data in CEUS images, yet research in this field is lacking. In our report, we found that PTL showed a rapid hyper enhancement pattern, with enlarged range than that of prey-scale US. These image features were consistent with the invasive growth pattern of testicular lymphoma. Lock et al. [17] analyzed the CEUS findings of seven testicular lymphoma lesions and found straight vessel pattern and rapid filling time were the CEUS characteristics of testicular lymphoma. After combining TIC quantitative analysis in our study, it was found that the TTP (s) was shorter than that of normal testis, while the Wash in slope (dB/s) was longer than that of normal testis. All these findings were similar to the previously reported [17] rapid filling time in CEUS. In addition, we also found that the PTL presented the characteristics of hyper enhancement with enlarged range. The reason for this characteristic might be related to the high expression of VEGF in malignant tumors, along with dysplasia of neovascularization forms arteriovenous anastomosis, which made the contrast medium passing quickly in the tumor and formed increased enhancement. On the other hand, the invasive growth pattern of testicular lymphoma caused the unclear boundary and enlarged lesion range in CEUS images. This phenomenon was similar to the numerous small echogenic spots with a speckled appearance in CEUS of lymph node in Non-hodgkin lymphoma reported by Rubaltelli [29]. However, whether these findings were the characteristic CEUS changes of testicular lymphoma remained to be confirmed by a multicenter large sample prospective study.

Our report was the first attempt to investigate the advanced US technique of microvascular image in PTL. As a noninvasive technique, microvascular imaging can detect slow and fine vascular flow, even the hepatic subcapsular [30]. As a malignant tumor of blood system, PTL has some particular pathological changes. An appearance of straight vessel sign was found to help diagnose PTL. In our report, a linear nonbranching vascular pattern on microvascular US was presented in PTL, which was similar to straight vessel sign.

## Conclusions

PTL is a kind of testicular malignant tumor with poor prognosis, while lacking an early non-invasive diagnosis. In this report, the CEUS images of PTL showed a hyper enhancement with enlarged range and unclear boundary along with a linear nonbranching pattern of vascular sign on microvascular US image. Combined with straight vascular signs on grey-scale US and painless testicular mass of physical examination, it can provide some help for early non-invasive diagnosis of PTL.

## Data Availability

The datasets will be available from the corresponding author upon reasonable request.

## Abbreviations

PTL: Primary testicular lymphoma
NHL: Non-Hodgkin's lymphoma
DLBCL: Diffuse large B-cell lymphoma
US: Ultrasonography
CEUS: Contrast-enhanced ultrasound
CDFI: Color Doppler flow imaging
TIC: Time-intensity curve
PI: Peak intensity
TTP: Time to peak
